# Acoustic pharyngometry ‐ A new method to facilitate oral appliance therapy

**DOI:** 10.1111/joor.13134

**Published:** 2020-12-28

**Authors:** Ulrik Leidland Opsahl, Morten Berge, Sverre Lehmann, Bjørn Bjorvatn, Per Opsahl, Anders Johansson

**Affiliations:** ^1^ Department of Clinical Dentistry ‐ Prosthodontics Faculty of Medicine University of Bergen Bergen Norway; ^2^ Norwegian Competence Center for Sleep Disorders Haukeland University Hospital Bergen Norway; ^3^ Tannhelsesenteret Lørenskog og Sogndal Lørenskog Norway; ^4^ Section for Thoracic Medicine Department of Clinical Science Faculty of Medicine University of Bergen Bergen Norway

**Keywords:** acoustic pharyngometry, oral appliance, sleep apnoea

## Abstract

**Background:**

There is lack of reliable and accurate methods to predict treatment outcomes of oral appliance (OA) treatment. Acoustic pharyngometry (AP) is a non‐invasive technique to evaluate the volume and minimal cross‐sectional area of the upper airway, which may prove useful to locate the optimal position of OAs.

**Objective:**

This retrospective study aimed to evaluate the effect of applying AP to OA treatment of patients with obstructive sleep apnoea (OSA).

**Methods:**

All patients (n = 244) treated with OAs following an AP protocol at two dental clinics between 2013 and 2018 were invited to participate. A total of 129 patients accepted the invitation, and 120 patients (75 men, 45 women) were included in the analyses. Mean baseline age, BMI and apnoea hypopnea index (AHI) were 59.1 ± 0.9 years, 27.8 ± 0.4 and 21.9 ± 1.1, respectively. Mean follow‐up time was 318 ± 24 days.

**Results:**

AHI at follow‐up was 6.4 ± 0.7, resulting in a treatment success rate of 86.7% (≥50% reduction of baseline AHI). The number of failures (<50% reduction of baseline AHI) did not differ significantly among patients with mild, moderate and severe OSA. 87.6% of the patients reported OA usage every night, and 95.5% reported > 5 hours usage per night, when worn.

**Conclusion:**

The AP protocol applied seems to contribute to the excellent effect of OA treatment in this study. Further research on the application of AP in OA treatment is necessary in order to clarify its possible beneficial contribution to improving OA therapy.

## 1 INTRODUCTION

Obstructive sleep apnoea (OSA) has a high prevalence in the general population with serious and widespread health implications.^1^ OSA is characterised by breathing cessations during sleep with simultaneous awakenings/arousals and reductions in arterial blood oxygen levels.^2^ In an adult Norwegian population, the prevalence was reported as 16% for mild OSA and 8% for moderate to severe OSA.^3^ However, a more recent study based on updated diagnostic criteria on a similarly aged Swiss population suggests even higher prevalence rates.^4^ Continuous positive airway pressure (CPAP) is the gold standard in OSA treatment, defined by criteria set by the American Association of Sleep Medicine (AASM),^1^ but the adherence to CPAP is generally poor, and reported as an average of 65.9% in a large meta‐analysis.^5^ Consequently, development of effective second‐line treatment options, such as oral appliance (OA) therapy, is crucial. In recent studies, excellent objective compliance with OA therapy has been reported.^6^, ^7^ Evidence suggests that OA treatment may be comparable to CPAP in improving health outcome, even in more severe OSA, as the greater usage of OA may compensate for its inferior efficacy compared to CPAP.^8^, ^9^ Objectively measured efficacy with OA treatment, that is reductions in apnoea hypopnea index (AHI) and oxygen desaturation index (ODI), will hardly ever reach that of CPAP. It would therefore be important to develop techniques in order to improve the efficacy of OA treatment.

OA titration/mandibular positioning is in many cases a ‘trial and error’ procedure, where the objective effect of each titration has to be assessed through an overnight polysomnographic (PSG) or an ambulatory polygraphic (PG) examination.^10^ Aside from complex and costly approaches, there is a lack of reliable and accurate methods to predict treatment outcome of OA therapy.^11^ Maximum comfortable protrusion is often referred to as the gold standard for titration of OAs, and clinicians often resort to increased mandibular protrusion when there is a lack of effect of OA treatment. However, a systematic review investigating the effectiveness of different mandibular advancements of OAs found that titration of the mandible above 50% of maximal protrusion did not significantly influence the success rate of the treatment, as measured by the reduction of AHI.^12^ A study investigating the minimal protrusion needed for sufficient effect of OA therapy concluded that a great proportion of the study sample showed sufficient AHI reduction with no protrusion at all.^13^ Historically, clinicians have relied on subjective symptoms as a measure of the efficacy of OA treatment. However, the relationship between an objective measure of the severity and subjective symptoms of OSA, such as daytime sleepiness, may be overestimated.^14^ A reduced effect of OA treatment often results in that the clinician increases the protrusion of the mandible by titrating the appliance, which may increase the risk of side effects,^15^ and which in turn could adversely affect the adherence to treatment.

The role of the vertical dimension in OA treatment remains unclear. Studies have shown that the vertical dimension of the appliance does not significantly impact the efficacy of the treatment,^16^, ^17^, ^18^ while another study found that increased vertical dimension in maximal comfortable protrusion might cause increased pharyngeal collapsibility during sleep.^19^ Nevertheless, the accepted gold standard for vertical dimension in OA treatment is to keep the vertical dimension as low as possible. Hence, the need for individualised mandibular advancement and vertical dimension of OA in order to optimise comfort and treatment efficacy seems obvious, and would be in accordance with recent consensus within the field of dental sleep medicine.^20^

### 1.1 Acoustic pharyngometry

**FIGURE 1:**
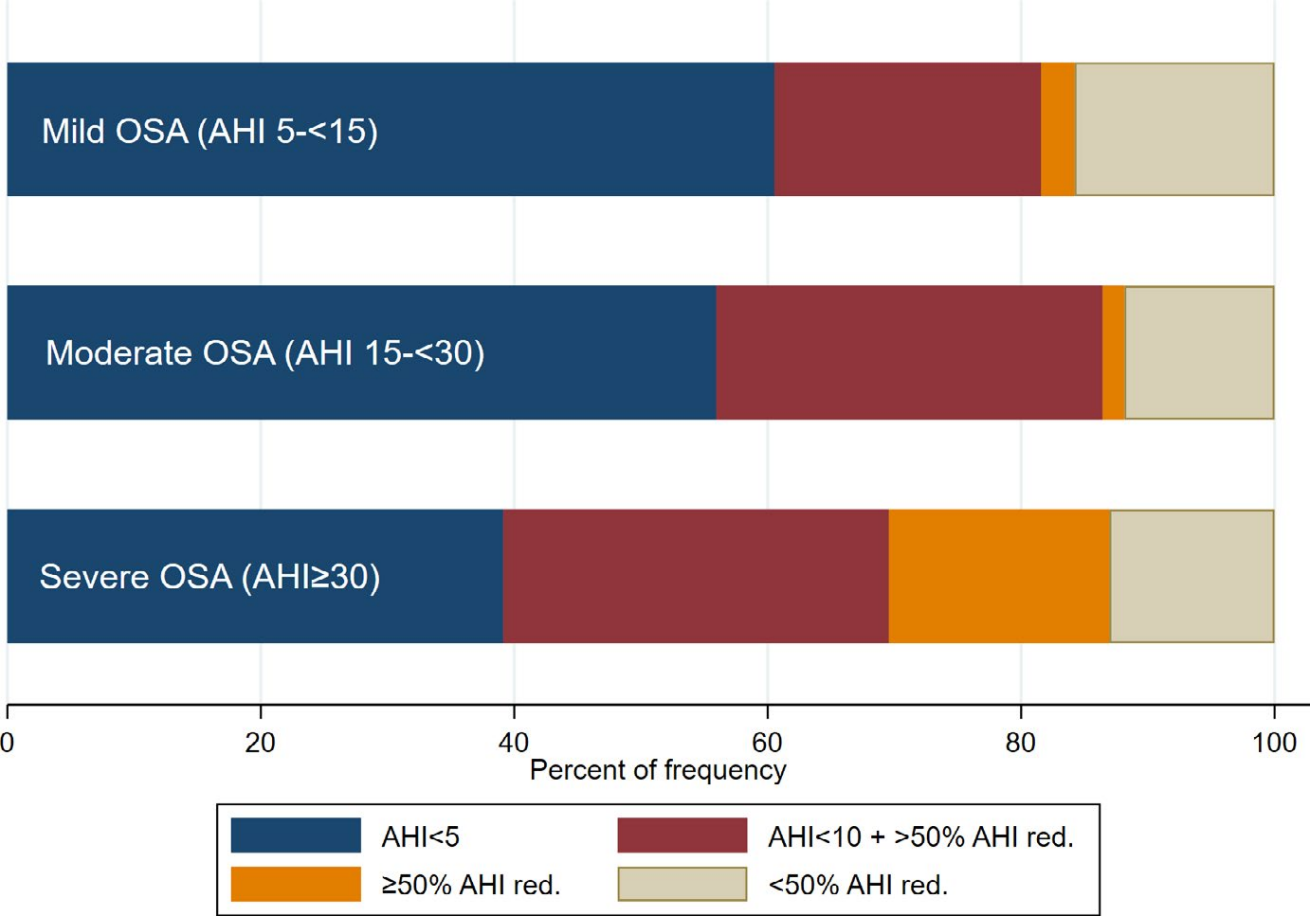
Comparison between baseline OSA diagnosis mild (n = 38), moderate (n = 59) and severe OSA (n = 23), according to success criteria applied at follow‐up after oral appliance treatment. Patients were only classified as one of the success criteria, being the lowest criteria possible; that is, if they achieved AHI < 10 and > 50% reduction of baseline AHI at follow‐up, the treatment would be classified as success criterion 2 and not success criterion 3 [Colour figure can be viewed at wileyonlinelibrary.com]

Acoustic pharyngometry (AP) is a non‐invasive technique that uses sound wave reflections to supply information about the cross‐sectional area and volume of the upper airway, from the lips to the epiglottis.^21^, ^22^ Information is graphically and instantly displayed as a curve representing the cross‐sectional area (y‐axis) along the first 25 cm of the upper airway (x‐axis) (Figure 1, Appendix 1). The total volume of the first 25 cm of the upper airway is depicted as the area under the curve for the sum of the cross‐sectional areas, while the minimal cross‐sectional area indicates where the upper airway is at its narrowest point. Airway volume measurements obtained with AP have been validated against computed tomography and magnetic resonance imaging, with acceptable accuracy.^23^, ^24^ The reproducibility of the AP registration technique has also been investigated.^25^, ^26^ A standard operating protocol for acoustic pharyngometry has been proposed for normal breathing, and the repeatability of the protocol has also been validated.^22^, ^25^

The collapsibility, or propensity for collapse of the upper airway (which is quantified as pressure differences in the upper airway during inspiration under negative generated pressure), and the minimal cross‐sectional area have been proposed to be predictors of the presence of moderate/severe OSA.^27^, ^28^ Volume changes of the pharyngeal cavity, as measured with AP, during positive and negative pressure indicate upper airway collapsibility, with significantly greater differences shown in OSA patients.^29^ Independent of age, gender and neck size, objective anatomical assessment with AP discriminates well between patients with mild versus moderate to severe OSA in a clinical setting.^30^ In addition, it has been shown that the pharynx of OSA patients shows greater tendency to collapse (i.e. collapsibility) when exhaling slowly from total lung capacity to residual volume (modified Mueller manoeuvre) compared with non‐OSA patients.^31^, ^32^ The collapsibility generated by the modified Mueller manoeuvre has previously been shown to be reproducible with acceptable intra‐class correlation (ICC).^33^ In regard to the foregoing, by using AP it should be possible to locate the position of the mandible that provides the lowest pharyngeal collapsibility and the largest minimal cross‐sectional area in the upper airway during a modified Mueller manoeuvre. This information could be used to determine the optimal position of the mandible and the OA.

The aim of this study was to evaluate the use of AP in OA treatment in patients with mild, moderate and severe OSA.

## 2 METHODS

### 2.1 Patients and diagnostic procedures

All patients (n = 244) referred to two dental clinics (125 to Tannhelsesenteret Lørenskog and 119 to Tannhelsesenteret Sogndal) for OA treatment between 2013 and 2018 were invited to participate in the study. The patients were treated according to AP protocol described in detail in Appendix 1. The treatment was performed by one of two calibrated dentists. The calibration comprised training sessions before the introduction of the AP methodology in the clinic. The calibration between the two dentists encompassed how to locate the optimal position of the OA based on the AP measures. A total of 129 patients (men = 80, women = 49) accepted the invitation and were included in the study. The baseline diagnosis of OSA was performed by ear, nose and throat specialists at referring hospitals, using one of two PG type IV devices (Embletta® or Nox T3®). The criteria for mild OSA were AHI 5 ‐ <15, for moderate OSA AHI 15 ‐ <30, and for severe OSA AHI ≥ 30.^1^ The objective effect of the OA treatment was investigated through home sleep testing with a PG type III device (Apnealink Plus^TM^ or Apnealink Air^TM^, ResMed Germany Inc), with scoring rules in accordance with the 2007 AASM guidelines.^34^ Two patients were excluded from the study because they were diagnosed using other scoring rules. A total of 7 patients were excluded from the study because they did not have an OSA diagnosis (AHI < 5 at baseline) and were treated with OAs on the indication of social snoring. The distribution of baseline diagnoses of the 120 patients finally included was the following: 38 patients mild OSA (32%), 59 moderate OSA (49%) and 23 with severe OSA (19%). A total of 111 patients (94%) had previously tried CPAP but were non‐compliant, 5 (4%) had never tried CPAP, and 2 (2%) of the patients did manage CPAP treatment but were referred for OA treatment for other reasons. Data describing previous CPAP compliance were missing for 2 patients.

### 2.2 Treatment protocol

The operating protocol for AP measures and locating the acoustic optimal position (AOP) of the mandible for the positioning of the OAs is described in detail in Appendix 1. Jaw registration was conducted in the individually determined position deemed optimal by AOP for all patients. Subsequent to delivery and adjustment of the OA, patients were instructed to use their OA throughout every night. The treatment was at minimum controlled after 3 weeks and 3 months. However, the majority of the patients were followed up for a longer time due to different follow‐up protocol required by referring hospitals in the early treatment years. Some patients were controlled more frequently if their OA needed adjustment to improve the effect of the treatment, or to handle compliance issues. PG registrations were performed at every follow‐up control, using the same PG type III device every time. The final visit to the clinic when the objective effect of the OA treatment was evaluated by PG registration was registered as the follow‐up date. Mean follow‐up time for the included patients was 318 days (95% CI: 271‐365 days).

The final follow‐up visit involved answering questionnaires regarding experienced side effects of the treatment, compliance and usage, subjective impact of the treatment on quality of life (QoL), subjective quality of sleep, snoring, daytime sleepiness and Epworth Sleepiness Scale (ESS, only recorded at follow‐up). The OA position was assessed with AP at each follow‐up control. If a better AOP compared to the current position was located, the OA was titrated to the new position. If patients reported a substantial positive effect on snoring, daytime sleepiness or other subjective OSA symptoms, and the PG recording showed adequate objective effect on AHI (AHI < 5), the OA was not adjusted from the baseline AOP, regardless of the new AP registration. Two different OAs were used in this study, the Narval CC^TM^ appliance (n = 88, 73%) and the Somnodent® Fusion (n = 32, 27%). The latter was used primarily in cases where the Narval appliance was not indicated due to retention issues or in cases where patients did not want the Narval appliance. None of the included patients used elastic bands on their OAs.

### 2.3 Success criteria

The success at the final PG follow‐up of the OA treatment was divided into three categories, according to Gjerde et al^34^ Patients were only classified according to one of the success criteria, being the lowest criteria possible; that is, if they achieved AHI < 10 and > 50% reduction of baseline AHI at follow‐up, the treatment would be classified as success criterion 2 and not success criterion 3.

- Success criterion 1: AHI < 5.
- Success criterion 2: AHI < 10 and > 50% reduction of baseline AHI.
- Success criterion 3: ≥50% reduction of baseline AHI.
- Failure: <50% reduction of AHI from baseline.

### 2.4 Statistical analyses

All statistical analyses were performed with Stata version 16.0 (College Station). Logistic regression analysis was performed in order to investigate associations with the strictest treatment success criterion 1 (AHI < 5) as the dependent variable. An identical logistic regression analysis was performed applying success criterion 3 (≥50% reduction of baseline AHI) as the dependent variable. Selection of independent variables in the unadjusted regression model included all those that were deemed to hypothetically influence the outcome of the dependent variable. Unadjusted logistic regression analysis (crude estimate) was first performed. Subsequently, an adjusted logistic regression analysis was performed including the independent variables exhibiting *P* < .1 in unadjusted analysis, applying a stepwise forward conditional method. Adjusted odds ratios with *P* < .05 were considered statistically significant in the adjusted model.

Unadjusted linear regression analysis was performed to investigate associations between side effects and subjective reported compliance. *P* < .05 was considered statistically significant in the linear regression. Kruskal‐Wallis test was used to analyse differences between baseline AHI in the three success groups and the failures. The Kruskal‐Wallis test was supplemented with a Mann‐Whitney *U* test to analyse significant differences using Bonferroni correction as Post hoc test, that is *P* < .017 and *P* < .008 for 3 and 4 groups, respectively, was considered statistically significant.

For the missing data analysis, linear regression analysis was performed to investigate differences in age, baseline BMI and AHI between the included respondents and the non‐respondents in this study. Pearson's chi‐squared test was performed to investigate differences in gender between respondents and non‐respondents. *P* < .05 was considered statistically significant.

All data are presented as mean ± standard error of the mean.

### 2.5 Ethics

The study was approved by the regional ethics committee of Western Norway, and informed written consent was obtained from all participants (protocol no: 2018/2121). The study was also approved by the Health and social services representative of the University of Bergen (protocol no: 2018/2121).

## 3 RESULTS

A total of 120 patients (men = 75, women = 45) were included in the follow‐up evaluation. At baseline, 16 patients (13.9%) were smokers, 28 (24.4%) had hypertension, 8 (7.0%) had cardiovascular disease and 3 (2.6%) diabetes. Table 1 provides further baseline patient characteristics. The overall success rate based on criteria 1 to 3 was 86.7%, and 81.7% based only on success criteria 1 to 2 (Figure 1). For all patients, mean AHI at follow‐up was 6.4 ± 0.7, a reduction from a mean AHI of 21.9 ± 1.1 at baseline, which represents a mean percentage AHI reduction of 66.7% ± 3.8. Follow‐up AHI in the three OSA baseline diagnoses of mild, moderate and severe, were 5.2, 5.6 and 10.3, respectively. When comparing the follow‐up AHI in the three OSA groups, the severe group had significantly higher AHI than the mild group (*P* = .01), but not so compared to the moderate group (*P* = .09) (Mann‐Whitney *U* test). There were no significant differences among the three baseline diagnoses and the success criteria recorded at follow‐up (Kruskal‐Wallis, *P* = .33).

**TABLE 1:** Baseline characteristics of included patients (n = 120)

	Mean	95% CI	SE	Min, max
**Demographic and clinical parameters**				
Age, y	59.1	57.4‐60.9	0.9	31, 77
BMI	27.8	27.1‐28.5	0.4	21, 38.8
Baseline AHI	21.9	19.6‐24.2	1.1	5.4, 73.8
VOB, mm	3.0	2.7‐3.3	0.2	0, 7
HOB, mm	2.9	2.5‐3.2	0.2	‐2, 8
Max interincisal distance, mm	47.1	46.1‐48.1	0.5	34, 62
Max protrusion, mm	9.5	9.3‐9.8	0.1	6, 13
Protrusion with OA, mm	6.7	6.3‐7.1	0.2	1, 13
Percentage OA protrusion of max	71.4	67.4‐75.5	0.2	12.5, 131.3
Interincisal distance with OA	5.6	5.3‐5.9	0.2	4, 12
Total vertical elevation with OA	8.4	8.0‐8.9	0.2	4, 16
Follow‐up time, days	318.2	271.4‐365.0	23.6	4, 158
**AP parameters**				
Mean baseline total UA volume, cm^3^	34.5	32.3‐36.7	1.1	5.1, 60.3
Mean collapse total UA volume, cm^3^	19.1	17.7‐20.4	0.7	3.7, 39.5
Mean AOP total UA volume, cm^3^	26.4	24.4‐28.4	1.0	6.9‐58.8
Mean AOPti total UA volume, cm^3^	27.3	25.2‐29.3	1.0	7.9, 52.3
Mean baseline min UA x‐sect area, cm^2^	2.1	2.0‐2.3	0.1	0.4, 7.5
Mean collapse min UA x‐sect area, cm^2^	1.1	1.0‐1.2	0.1	0.2‐3.2
Mean AOP min UA x‐sect area, cm^2^	1.6	1.5‐1.8	0.1	0.5, 3.4
Mean AOPti min UA x‐sect area, cm^2^	1.8	1.7‐2.0	0.1	0.5, 4.3

Note

Abbreviations: SE, standard error, CI, confidence interval for variable, VOB, vertical overbite, HOB, horizontal overjet, OA, oral appliance, AP, acoustic pharyngometry, UA, upper airway, x‐sect, cross‐sectional, AOP, acoustic optimal position, AOPti, oral appliance in the final position after any necessary titration.

**FIGURE 2:**
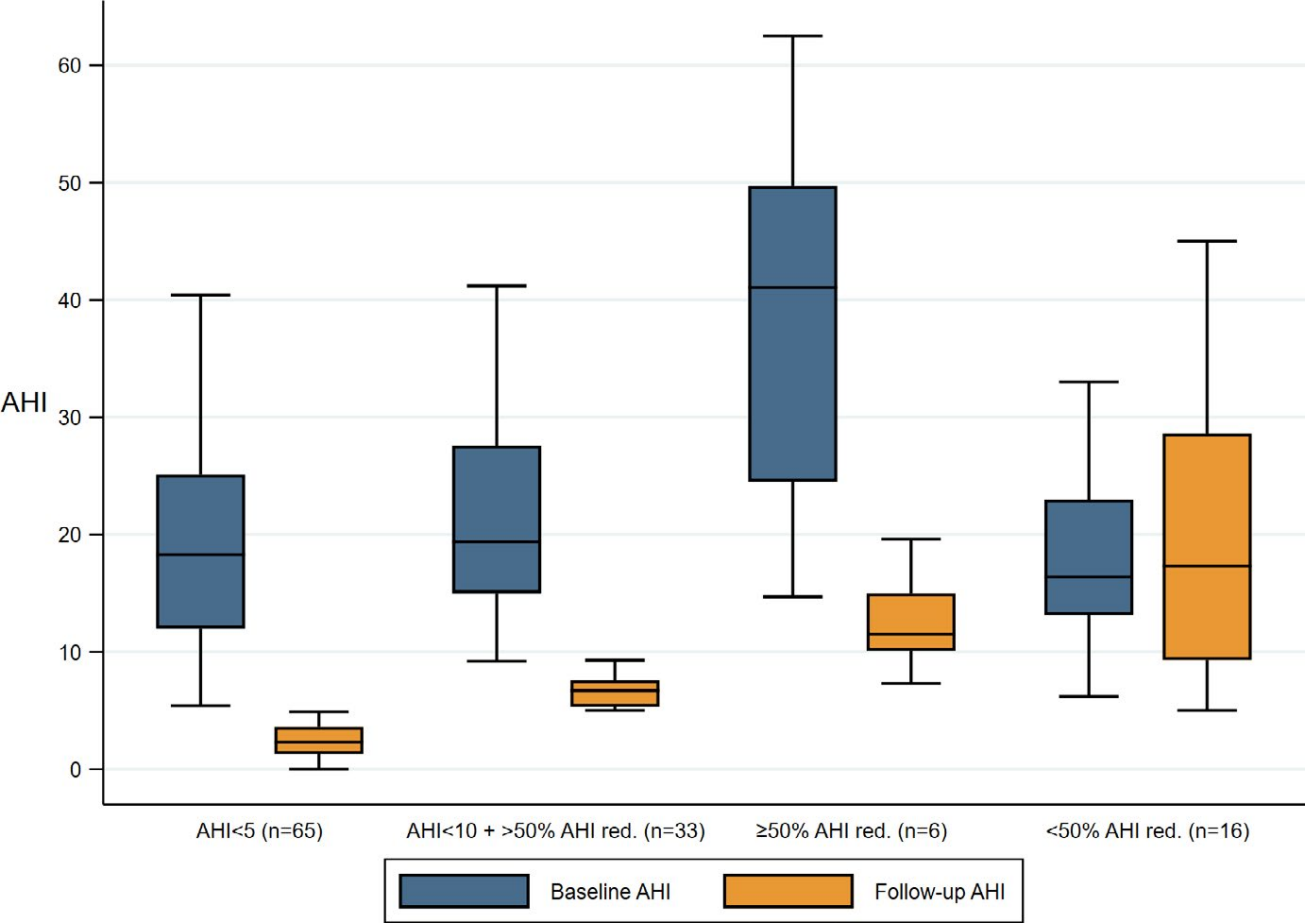
Distribution of baseline and follow‐up AHI between patients, grouped after success criteria obtained at follow‐up [Colour figure can be viewed at wileyonlinelibrary.com]

Baseline AHI was significantly different among patients obtaining success criteria 1, 2, and 3 and the failures at follow‐up (Kruskal‐Wallis, *P* = .0001) (Figure 2). This difference was attributed to patients achieving success criterion 3 (≥50 reduction of baseline AHI) compared criterion 1 (AHI < 5) at follow‐up (*P* = .006), i.e. significantly higher baseline AHI in those who reached criterion 3 at follow‐up. Bivariate group comparisons between the other categories and baseline AHI revealed no significant differences when applying Bonferroni correction.

Mean ODI at follow‐up was 6.7 ± 0.7, mean SaO_2_ was 92.7% ± 0.1%, and mean percentage of time with SaO_2_ < 90% saturation was 14.3% ± 2.0%. Mean ESS at follow‐up was 6.1 ± 0.4.

As regards self‐reported compliance at follow‐up, 87.6% of the patients reported usage of their OA every night, and 95.5% reported > 5 hours usage per night, when worn. Of the 87.6% that reported usage of their OA every night, 95.9% reported > 5 hours usage per night. Improved QoL at follow‐up was reported by 88.4% and no change by 11.6%. None of the patients reported worsening of QoL. Excellent or adequate subjective effect on snoring was reported by 81.2%, 78.2% on quality of sleep, and 75.7% on daytime sleepiness.

Regarding side effects, 14.9% of the patients responded in the affirmative to sounds/pain from the temporomandibular joints, 17.5% to pain or tension from masticatory muscles, 9.7% headache, 14.9% hypersalivation and 43.9% dry mouth. These figures do not necessarily represent the status at follow‐up but reflect side effects occurring during the course of OA treatment, and were often diminishing in nature. Reported pain/tension in the masticatory muscles correlated significantly with reduced compliance, inferred from how many nights per week the OA was used (*P* < .001, unadjusted linear regression analysis). None of the other side effects showed any significant correlations with OA compliance. Interincisal distance with the OA in the AOP was 4 mm in 43.0% of the patients and > 4 mm for the remaining patients (57.0%). The distribution was 6 mm (38.6%), 8 mm (14.0%), 10 mm (3.5%) and 12 mm (0.9%) interincisal distance.

### 3.1 The failures

In total, 16 patients (13.3%) were classified as failures (<50% reduction of AHI from baseline). The failures had a mean baseline AHI of 19.5 ± 2.6 and a mean AHI of 19.8 ± 3.3 at follow‐up. In the adjusted logistic regression model, reported hypertension and smaller increase in total vertical elevation (overbite + vertical thickness of OA) were predictors of failure (Table 2). The mean total increases of vertical dimension with the OA were 6.6 ± 0.5 mm (CI: 5.6‐7.7 mm) and 8.7 ± 0.3 mm (CI: 8.2‐9.2 mm) in the failure group and the three success groups combined, respectively.

**TABLE 2:** Logistic regression model for the odds of failure of OA treatment (<50% reduction of AHI from baseline) as dependent variable for the included 120 patients in the study. Dependent variable dichotomised as 0 = ≥50% reduction of AHI from baseline, 1 = <50% reduction of AHI from baseline

Reference category	Unadjusted			Adjusted		
	OR	95% CI	*P*	OR	95% CI	*P*
AHI baseline	0.98	0.93‐1.03	NS	‐	‐	‐
BMI	1.17	1.03‐1.34	**	‐	‐	NS
Gender	1.00	0.34‐2.97	NS	‐	‐	‐
Age	1.05	0.99‐1.11	*	‐	‐	NS
No hypertension	0.30	0.10‐0.93	**	0.25	0.07‐0.86	**
No CHD	0.21	0.04‐0.99	**	‐	‐	NS
Total vertical elevation	0.68	0.52‐0.89	***	0.64	0.48‐0.87	***
No diabetes	0.29	0.02‐3.36	NS	‐	‐	‐
HOB	0.89	0.65‐1.21	NS	‐	‐	‐
% of max protrusion	0.96	0.07‐14.00	NS	‐	‐	‐
Type of OA	1.63	0.57‐4.61	NS	‐	‐	‐
All AP measures			NS	‐	‐	‐

Note

Abbreviations: OR, odds ratio, CI, confidence interval for OR, CHD, coronary heart disease, OA, oral appliance, VOB, vertical overbite, HOB, horizontal overjet, AP, acoustic pharyngometry.

Prob > chi^2^ = 0.0005; Pseudo *R* ^2^ = 0.182.

**** *P* ≤ .001; ^***^.001 < *P* ≤ .01; ^**^.01 < *P* ≤ .05; ^*^.05 < *P* ≤ .1.

None of the variables commonly reported to be associated with success of OA treatment, such as gender, age and amount of protrusion, turned out to be significant predictors. Moreover, type of OA, subjectively reported compliance, or any of the AP measures were not significantly associated with failure of OA treatment (data not shown in tables).

Three patients showed higher AHI with OA compared to baseline AHI. In those patients, a new PG recording was performed without the OA and the results showed an aggravation of the OSA compared to that at the referral time. Still, in those three patients, we used the original baseline diagnosis and AHI in the analyses, and all of them consequently became treatment failures.

### 3.2 Acoustic pharyngometry results

Descriptive data for AP measurements are shown in Table 1. No adjustments (protrusive titration) of the OA were needed in 18 (15.0%) of the patients, while 72 (60.0%) needed a few adjustments, and 23 (19.2%) needed several adjustments. OAs were adjusted if: (a) objective efficacy was suboptimal (AHI > 5) and AP measures displayed a new AOP; (b) a gradual titration towards AOP was indicated due to side effects; (c) patients experienced lack of retention of their OAs; or (d) the OA caused ulcerations and was adjusted in order to relieve the ulcerated area. Hence, how many of the OAs that needed titration exclusively to optimise objective efficacy was not recorded. For 7 patients (5.8%), new OAs were fabricated either due to lack of retention, suboptimal efficacy and AOP with a different vertical dimension, or due to lack of compliance with the current type of OA.

The percentage decrease in total upper airway volume from the baseline registration (functional residual volume, mean total volume 34.5 cm^3^) compared to the collapse registration (residual volume generated by modified Mueller manoeuvre, mean total volume 19.1 cm^3^) correlated significantly with higher baseline AHI (linear regression, *P* < .05). Hence, patients demonstrating a more collapsible airway also had a more severe OSA diagnosis at baseline.

In the unadjusted logistic regression analysis, several variables were significantly correlated with achieving the strictest success criterion at follow‐up (AHI < 5). However, in the adjusted model only reported absence of hypertension, lower BMI and greater AP total volume maintained during collapse registration at baseline were significantly correlated with achieving success criterion 1 (AHI < 5) at follow‐up (OR 2.90, OR 0.88, and OR 1.07, respectively) (Table 3).

**TABLE 3:** Logistic regression model for the odds of achieving success criterion 1 after OA treatment (AHI < 5), with success criterion 1 as dependent variable for the included 120 patients in the study. Dependent variable dichotomised as 0 = AHI ≥ 5, 1 = AHI < 5

Reference category	Unadjusted			Adjusted		
	OR	95% CI	*P*	OR	95% CI	*P*
AHI baseline	0.98	0.95‐1.00	NS	‐	‐	NS
BMI	0.83	0.75‐0.93	****	0.88	0.78‐0.98	**
Gender	1.46	0.69‐3.09	NS	‐	‐	‐
Age	0.99	0.95‐1.02	NS	‐	‐	‐
No hypertension	3.29	1.33‐8.11	***	2.90	1.03‐8.12	**
No CHD	11.57	1.40‐95.6	**	‐	‐	NS
Total vertical elevation	1.11	0.95‐1.28	NS	‐	‐	‐
No diabetes	2.39	0.21‐27.15	NS	‐	‐	‐
VOB	1.25	1.00‐1.57	*	‐	‐	NS
HOB	1.00	0.82‐1.22	NS	‐	‐	‐
% of max protrusion	0.30	0.05‐1.75	NS	‐	‐	‐
Type of OA	0.87	0.40‐1.89	NS	‐	‐	‐
Vertical dim. OA	1.00	0.81‐1.24	NS	‐	‐	‐
AP min cross‐sectional area AOP	1.67	0.91‐3.06	*	‐	‐	NS
AP total volume collapse	1.05	1.00‐1.10	*	1.07	1.01‐1.13	**

Note

Abbreviations: AOP, acoustic optimal position;AP, acoustic pharyngometry; CHD, coronary heart disease; CI, confidence interval for OR; HOB, horizontal overjet; OA, oral appliance; OR, odds ratio; VOB, vertical overbite.

Prob > chi^2^ = 0.0010, Pseudo *R* ^2^ = 0.1183.

**** *P* ≤ .001; ***.001 < *P* ≤ .01; **.01 < *P* ≤ .05; *.05 < *P* ≤ .1.

Unadjusted linear regression analysis showed no significant correlations between AHI at follow‐up and subjective reported outcome variables, such as ESS, subjective reported compliance, change in QoL, daytime somnolence, snoring and perceived quality of sleep (data not shown in tables).

### 3.3 Missing data analysis

Of the total 244 patients, 120 out of the 129 respondents were included in the final analysis, while 115 (non‐respondents) did not give informed consent and were not included. Missing data analysis was performed between the respondents and non‐respondents regarding age, gender, baseline BMI and AHI. There were no significant differences between respondents and non‐respondents with regard to mean baseline AHI (21.9 vs 21.7) and mean BMI (27.8 vs 28.6), while age (59.1 years (respondents) vs 52.9 years (non‐respondents); *P* < .001) and gender (62.5% men / 37.5% women (respondents) vs 75.2% men / 24.8% women (non‐respondents); *P* < .05) differed significantly.

## 4 DISCUSSION

This retrospective study of 120 OSA patients treated with OAs following an acoustic pharyngometry protocol showed an overall success rate of 86.7% using success criterion 1‐3 (≥50% reduction of AHI from baseline). When analysing patients with moderate (n = 59) and severe (n = 23) OSA separately, the success rates using criterion 1‐3 were 88.1% and 87.0%, respectively, which indicates that the success rate for this population was not affected by OSA severity (Figure 1). The result of OA treatment following the AP protocol applied in this study seems to be superior compared to studies following the standard protocol for OA treatment using similar success criteria (≥50% reduction of AHI from baseline), where success rates of 77% and 69% were achieved for patients with moderate and severe OSA respectively in one study, and a success rate of 58% for the entire population of mild to severe OSA patients in another.^35^, ^36^ A more recent study describing outcomes of standard protocol OA treatment in severe OSA patients presented 60% success following success criteria 3.^37^ The patients included who were treated with an effective OA and not a sham device (n = 55) had comparable baseline AHI, BMI, occurrence of hypertension and distribution of men/woman as the patients with severe OSA (n = 23) in this study. However, the difference in number of included patients should be noted.

The missing data analysis showed that the included patients were representative for the population in terms of baseline AHI and BMI, while age and gender differed significantly between the respondents and non‐respondents; that is, the non‐respondents had an overrepresentation of men and younger individuals. Younger age has previously been identified as a predictor for response to OA treatment (≥50% reduction of baseline AHI).^38^ This finding is deemed to be a positive bias, indicating that the treatment effect for the entire group might be better than that reported for the patients in our study group. Further, female gender has been shown to predict treatment success in one report,^39^ while it did not in another study.^38^ Based on these reports, it is difficult to draw any conclusion as to whether the overrepresentation of women in the study group constituted a bias or not.

The AP protocol aims for individualisation of OA treatment. Standard protocol OA treatment suggests ‘maximal comfortable protrusion’, which often results in 50%‐80% of maximal protrusive range as the target OA position for optimal treatment effect.^40^, ^41^ A systematic review investigating clinical practice in OA titration found that all included studies describing percentage of maximal protrusion reported a mean protrusion exceeding 85%.^10^ ‘Maximal comfortable protrusion’ was not recorded in the present study but the mean of 71.7% of maximal protrusion for the OAs indicates that the OAs were less protruded than what is commonly applied. Regarding individualisation of OA treatment, a recently published study investigating target protrusion of OAs after stepwise titration with PG registration at each protrusive adjustment found that the target protrusion increased with increasing OSA severity, ranging from 38.6% to 68.8% of maximal protrusion.^42^

In a recent systematic review and meta‐analysis, several phenotypes predicting response to OA treatment were identified.^43^ In our study, low BMI and absence of hypertension could predict treatment success in analogy to these findings. In addition, we found that increased total vertical elevation introduced by the OA could significantly predict treatment success. The total increase of vertical interincisal dimension of the OAs ranged from 4 to 12 mm, with a mean of 5.6 mm. A recent study identified patients with larger overbite as significantly more likely to achieve complete response of OA treatment, hypothesising that increased vertical dimensions for these patients normalises the intermaxillary space for the tongue in the oral cavity.^44^ However, a general conclusion should not be drawn that increased vertical dimension improves the effect of OA treatment. The message is that horizontal and vertical individualisation of the OA position by using AP seems to improve the treatment effect. If the vertical dimension plays an important role in OA treatment, it is evident that locating the individualised optimal vertical (and protrusive) position would not be possible following standard protocol for OA treatment. In addition, presently available standard OAs do not allow adjustment of the vertical dimension. Accordingly, AP seems to be an efficient way to locate the individualised optimal combined vertical and horizontal position of OAs.

The collapsibility of the upper airway, displayed as the reduction in total volume from the baseline to the collapse registration (modified Mueller manoeuvre) measured with AP, correlated significantly with OSA severity at baseline, which seems to be logical as patients with severe OSA probably have more collapsible upper airways than those with less severe disease. This has also been demonstrated in previous studies with AP.^31^, ^32^ In our study, larger minimum cross‐sectional area and total volume maintained in the AOP, and larger AP total volume during collapse, showed indications of being predictors of reaching success criterion 1 (AHI < 5) with OA treatment in the unadjusted model. In the adjusted model, however, only total volume maintained during collapse registration at baseline remained a significant predictor for reaching success criterion 1. The latter finding indicates that a larger total volume maintained during collapse registration, indicating a less collapsible airway, increased the odds for reaching success criterion 1. Although it should be noted the odds ratio was only 1.07 for this prediction, so the clinical benefit of this should be interpreted with caution. Nevertheless, based on the above findings, the applicability of AP in OA treatment deserves further attention and more in‐depth studies, which are currently underway in our research group.

The occurrence of reported side effects was rather significant in this study population, especially for dry mouth (43.9%). However, the reported side effects did not exceed that found in other studies.^45^, ^46^ When reporting side effects, patients were asked if they had experienced any of the listed side effects in association with OA treatment. Hence, some patients might have reported the side effects commonly occurring in the initial phase of OA treatment which usually disappear after a relatively short time. Further, other than pain/tension in the masticatory muscles, there was no significant relationship between side effects and reported usage of the OA, indicating that the side effects did not affect treatment compliance in this population.

### 4.1 Limitations

The uncertainty of the reproducibility and validity of AP measures is a limitation of the technique. The results of the treatment indicate that AP improves OA treatment compared to standard OA treatment, when performed by two calibrated and experienced clinicians. The current study was, however, not performed with a randomised design including a control group, and direct comparison with other studies is not possible due the differences in case selection, etc. The clinicians performing the AP and the positioning of OAs in this study were highly skilled in using the technique, and the results cannot directly be extrapolated in the hands of lesser experienced dentists. This will require further investigations. There is also an uncertainty regarding the reproducibility of the modified Mueller manoeuvre, and the fact that the measures are performed awake and in a seated position. The rationale for using the modified Mueller manoeuvre when demonstrating collapsibility of the upper airway is that this technique was previously shown to demonstrate a reduction in pharyngeal cross‐sectional area in OSA patients, which was not present in non‐OSA subjects.^31^, ^32^ As the subjects in above‐mentioned studies were seated and awake during AP measures, this was applied in our protocol as well (Appendix 1). The reproducibility of total lung capacity and residual volume has previously been shown to have acceptable ICC.^33^

Some of the included patients were diagnosed with OSA some years prior to the OA treatment. This means that, in some patients, the true AHI at baseline might have been different at the start of OA treatment, due to, for example, greater age and changed BMI.^4^ This was possibly the case in three patients where the initial follow‐up AHI with OA was higher than the baseline AHI. In those patients, a new PG recording was performed without the OA and the results showed an aggravation of the OSA diagnosis compared to that at the referral time. Still, in those three patients, we used the original baseline diagnosis and AHI in the analyses, and all of them consequently became treatment failures, as reported in the results. Nonetheless, this uncertainty would in most cases only generate false‐negative rather than false‐positive results, due to the unlikelihood of reduced AHI in the absence of intensive lifestyle interventions.^47^

There is also some uncertainty about using different diagnostic devices for baseline and follow‐up sleep recordings. All follow‐up registrations were made with the same type III devices. These devices have previously shown comparable accuracy in diagnosing OSA with a type IV device (Embletta®)^48^ and with PSG.^49^, ^50^ The type III devices used have shown a slight tendency to overestimate AHI compared to Embletta® at lower values, when using autoscore,^48^ but this would be correctly adjusted as the present type III reports were manually scored in this study, and would also, if AHI was overestimated, result in false‐negative results.

In summary, the present results indicate that OA treatment following an AP protocol provides high treatment success rates in the hands of trained clinicians, applying considerable variation in the vertical dimension for OAs, ranging from 4 to 12 mm. Individualisation of OA treatment through locating the AOP seems to play a vital role in improving the treatment outcome. However, further research to investigate the possible health and cost benefits of applying AP in OA treatment is needed to broaden the current findings.

## 5 CONCLUSION

The AP protocol applied in this study seems to provide a very high OA treatment success rate. Further research on the application of AP in OA treatment is needed in order to clarify its likely beneficial contribution to improving OA therapy.

## CONFLICT OF INTEREST

We declare no conflict of interest.

## AUTHOR CONTRIBUTIONS

All authors contributed to concept and study design. Authors PO and ULO contributed to data collection. All authors contributed to interpretation of results and critical revision of the manuscript, and provided final approval before submission.

### Peer Review

The peer review history for this article is available at https://publons.com/publon/10.1111/joor.13134.

## Figures and Tables

**FIGURE A1:**
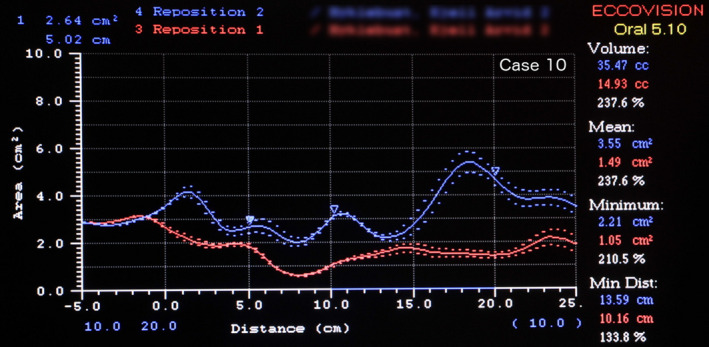
An example of acoustic pharyngometry data for baseline and collapse registrations displayed in the same graph. The cross‐sectional area is displayed on the y‐axis (Area, cm^2^) and the distance displayed on the x‐axis (Distance, cm), measured from the lips at 0.0 cm and 25.0 cm into the upper airway. The column to the right displays the results of the measures, showing the total volume (Volume), the mean cross‐sectional area (Mean), the minimal cross‐sectional area (Minimum) and the distance from the lips to the minimal cross‐sectional area (Min Dist) [Colour figure can be viewed at wileyonlinelibrary.com]

**FIGURE A2:**
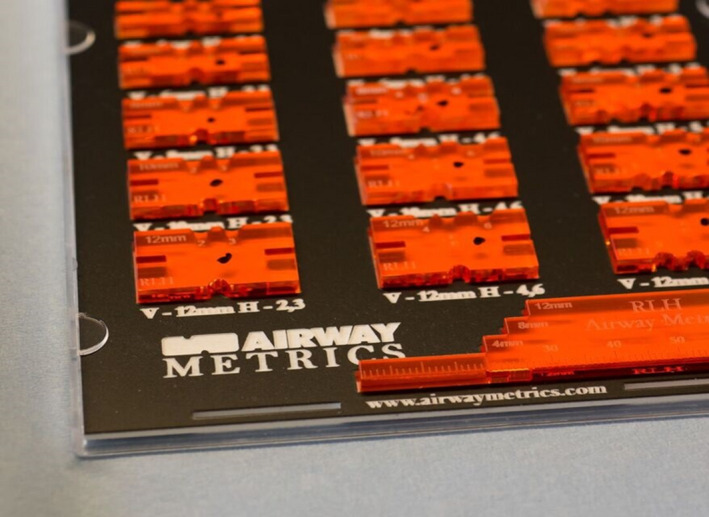
The airway metrics system displayed, with bite‐jigs with various thicknesses and notches on both sides of the jig, allowing the clinician to place the patient's mandible in different horizontal and vertical positions in a controlled manner [Colour figure can be viewed at wileyonlinelibrary.com]

**FIGURE A3:**
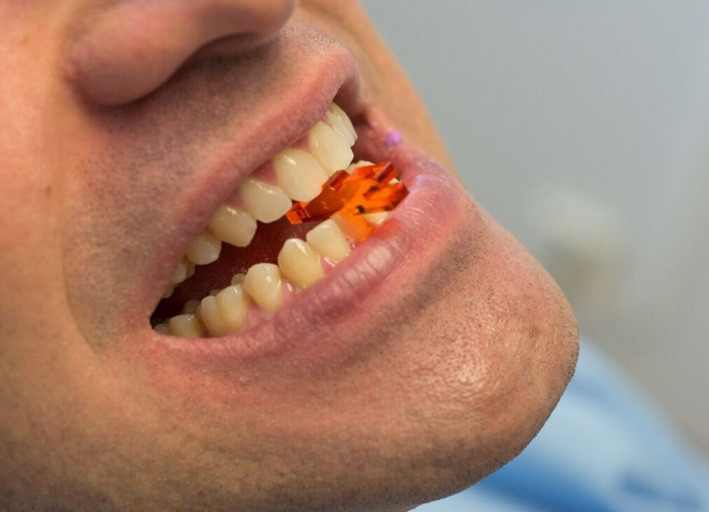
An example of how to use the bite jigs in the airway metrics system. This patient's mandible is positioned with 4 mm interincisal distance and 2 mm protrusion from edge to edge [Colour figure can be viewed at wileyonlinelibrary.com]

**FIGURE A4:**
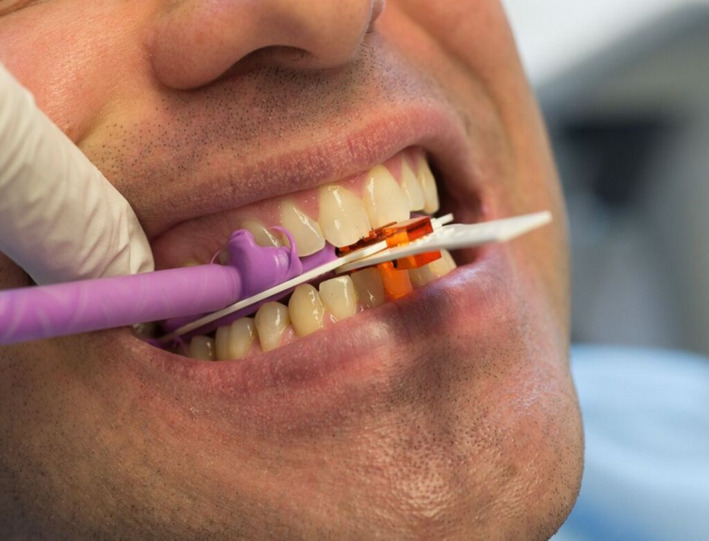
Jaw registration is performed in the AOP using the bite jig that provides the corresponding horizontal and vertical position, which is 4 mm interincisal distance and 2 mm protrusion from edge to edge in this example. A bite fork and handle are fitted into opposite ends of the bite jig, providing stability for the jaw registration material, which is added in a controlled manner while the patient is positioned in the AOP [Colour figure can be viewed at wileyonlinelibrary.com]

## Data Availability

Data can be made available upon request.

## APPENDIX 1 Acoustic pharyngometry ‐ A new method to facilitate oral appliance therapy

All patients treated with oral appliances (OAs) at the two dental clinics at Tannhelsesenteret Lørenskog and Tannhelsesenteret Sogndal between 2013 and 2018 were treated according to the following protocol, referred to as an acoustic pharyngometry (AP) protocol. This protocol is based upon the standard operating protocol for acoustic pharyngometry previously described by Kamal.^25^

**General information about acoustic pharyngometry examination**

1. *Patient position:* The patient is seated in an upright position on a straight‐back chair and instructed to maintain a neutral head position. The patient's comfort was enabled by positioning the chair with its back against a wall, so that their head could be supported/stabilised by contact with the wall. The patient is instructed to remain still during the examination and to fix their eyes on something at the same height on the opposite side of the room, thus keeping the head in an unchanged horizontal position.
2. *Patient considerations:* Patients were told to silently pronounce the word ‘ooh’ to rest their tongue in a relaxed position on the floor of the mouth, and to compress their nostrils (by pinching with thumb and forefinger) so as to prevent air from escaping, throughout the examination (Eccovision acoustic pharyngometry manual).
3. *Wave tube and mouthpiece:* The snorkel‐like mouthpiece is made of rubber and is designed with a flange under the lips to form an acoustic seal (Eccovision acoustic pharyngometry manual). Sound waves, which are produced by the wave tube, are introduced into the oral and pharyngeal cavities via the mouthpiece and reflected back to a microphone housed in the wave tube. The wave tube is placed horizontally, parallel to the floor (Eccovision acoustic pharyngometry manual).
4. *The baseline registration:* The baseline registration is measured at functional residual capacity (FRC). The patient is instructed to slowly inhale and exhale, similar to normal breathing. AP data are registered at the end of normal expiration.
5. *The collapse registration:* The collapse registration is measured at residual volume (RV). RV is generated using the modified Mueller manoeuvre, in which the patient slowly exhales from total lung capacity (TLC) to RV. The patient is first informed about the modified Mueller manoeuvre and practices it together with the clinician, who remains in attendance until after the manoeuvre is accomplished. The rationale for using the modified Mueller manoeuvre is that OSA patients have previously been shown to be more prone to have a more collapsible airway at RV than subjects without OSA.^31^, ^51^

Figure A1 is an example of AP data for baseline and collapse registrations displayed in the same graph.

**Locating the acoustic optimal position**

1. First, baseline and collapse registration are performed when biting on the mouthpiece in habitual occlusion to establish the baseline values. Because of the thickness of the rubber mouthpiece, the baseline registrations are performed with 2 mm interincisal distance.
2. The airway metrics system (Figure A2) provides bite jigs with various thicknesses and notches on both sides of the jig, allowing the clinician to place the patient's mandible in different horizontal and vertical positions in a controlled manner (Figure A3). Collapse registrations are sequentially performed with the mandible in different horizontal and vertical positions, starting at 50% of maximal protrusion and 4 mm interincisal distance. Next, AP measures are performed at the same 4 mm interincisal distance, but at both increased and reduced protrusion. Further, identical procedures are performed with 2 mm incremental increases in interincisal distance, until all five interincisal distances, from 4 to 12 mm, are investigated. If a trend of reduced upper airway volume with increased interincisal distance is observed, further investigations with increased vertical position are not performed. Similarly, if increased or decreased protrusion at a fixed interincisal distance results in reduced upper airway volume, further investigation in the given horizontal direction is not performed. The position demonstrating the largest total volume and largest minimal cross‐sectional area during collapse registration is deemed the acoustic optimal position (AOP). If two or more positions show similar AP values, the position with the least protrusion is chosen due to lesser potential side effects.^15^ If two positions with equal protrusion and different interincisal distances show similar AP values, the position with the lowest interincisal distance is chosen, again for better patient comfort, thus hypothetically providing better compliance with the treatment.

**Jaw registration**

1. Jaw registration is performed in the AOP using the bite jig that provides the corresponding horizontal and vertical position. A bite fork and handle are fitted into opposite ends of the bite jig, providing stability for the jaw registration material (Figure A4). The jaw registration, once cured, ensures that the registration is performed in the intended horizontal and vertical position.

**Delivery and control of oral appliances**

1. Upon delivery and at each follow‐up of the OA, collapse registrations are performed with the OA in place, first in the AOP, and thereafter at a 1 mm protruded, and a 1 mm retruded position relative to the AOP. If a trend of greater volume and maintenance of minimal cross‐sectional is discovered, this position is investigated further by protruding/retruding the OA with successive 1 mm increments, while performing new collapse‐registrations at each horizontal position of the OA. If a new AOP is discovered, the OA is adjusted to this position.
2. If, before follow‐up, the patient reaches success criterion 1 (AHI < 5) with the OA in the current AOP, control of the OA with AP is not performed.
3. If the patient experiences significant side effects, resulting in compliance‐issues, the OA is retruded to a position that is acceptable to the patient, and then gradually titrated forward to the previously determined AOP at subsequent follow‐ups.
4. If patients have problems with retention of the OA, or other problems related to the type of OA, another type of OA is made in the same AOP as the current OA.
